# The association of diabetes, subclinical hypothyroidism and carotid intima-media thickness: results from the Brazilian Longitudinal Study of Adult Health (ELSA-Brazil)

**DOI:** 10.1016/j.clinsp.2022.100154

**Published:** 2023-01-19

**Authors:** Aída de Melo Spilack, Alessandra C. Goulart, Bianca de Almeida-Pititto, Carolina Castro Porto Silva Janovsky, Paulo A. Lotufo, Itamar de Souza Santos, Isabela M. Benseñor

**Affiliations:** a Post-Graduate Student, School of Medicine, Universidade de São Paulo, São Paulo, SP, Brazil; b Centro de Pesquisa Clínica e Epidemiológica, Hospital Universitário, Universidade de São Paulo, São Paulo, SP, Brazil; c Departamento de Epidemiologia, Faculdade de Saúde Pública, Universidade de São Paulo, São Paulo, São Paulo, SP, Brasil; d Programa de Pós-Graduação em Epidemiologia, Universidade Federal do Rio Grande do Sul, Rio Grande do Sul, Porto Alegre, RS, Brasil

**Keywords:** Carotid intima-media thickness, Diabetes mellitus, Subclinical hypothyroidism, Subclinical atherosclerosis, Cardiovascular disease

## Abstract

**Introduction:**

The association of diabetes with subclinical thyroid diseases may increase the risk of cardiovascular diseases. We analyzed the association of subclinical hypothyroidism, diabetes, and both diseases with carotid Intima-Media Thickness (cIMT) as a surrogate maker for early cardiovascular disease in the Brazilian Longitudinal Study of Adult Health (ELSA-Brasil).

**Methods:**

Cross-sectional analysis with data from the 3^rd^ visit (2017‒2019). Linear regression models were used to evaluate the association of subclinical hypothyroidism, diabetes and of both diseases with a cIMT presented as Beta (95% Confidence Interval ‒ 95% CI) without adjustment, with adjustment for sociodemographic variables (Model 1) and multivariable adjustment (Model 1 more cardiovascular risk factors). We also used logistic regression models to analyze the Odds Ratio (OR) and 95% CI for the association of both diseases using cIMT > P75%.

**Results:**

After the exclusion of patients with previous cardiovascular disease, 5,077 participants with no diseases, 1578 with diabetes, 662 with subclinical hypothyroidism, and 234 with both diseases were included in the analysis. Linear regression models showed an association of cIMT with only diabetes (β = 0.019; 95% CI 0.012 to 0.027; p < 0.0001) and subclinical hypothyroidism more diabetes (β = 0.03; 95% CI 0.010‒0.047, p < 0.0001). The logistic regression model reported an association between diabetes and CIMT higher than P75% (OR = 1.49, 95% CI 1.30‒1.71). No interaction between diabetes and subclinical hypothyroidism was detected using cIMT respectively as a continuous (p = 0.29) or as a categorical variable (p = 0.92).

**Discussion:**

Diabetes was associated with higher cIMT values. However, no additive effect of subclinical hypothyroidism associated with diabetes over cIMT was detected.

## Highlights

• cIMT is associated with diabetes.
• Subclinical hypothyroidism was not associated with cIMT.
• No additive effect over cIMT was detected in patients with both diseases.

## Introduction

It has been estimated more than 400 million adults aged 20‒79 years living with diabetes in 2019 with alarming statistics revealing about 700 million adults by 2045.^1^ Thyroid dysfunction are frequent diseases with high prevalence of overt and subclinical hypothyroidism worldwide.^2^

The association of diabetes and overt hypothyroidism is very frequent suggesting that it might not occur by chance.^3^ Some studies also showed high prevalence of subclinical hypothyroidism^4^, ^5^, ^6^ and less frequently of subclinical hyperthyroidism in patients with type 2 diabetes.^5^^,^^7^ This association is probably bidirectional with a higher frequency of diabetes in patients with overt^8^^,^^9^ or subclinical hypothyroidism.^8^ In addition, some studies showed that complications of diabetes such as Coronary Heart Disease (CHD), chronic kidney disease, peripheral arterial disease and diabetic retinopathy are more frequent in patients with both type 2 diabetes and subclinical hypothyroidism.^6^^,^^10^^,^^11^

Moreover, diabetes^12^ and subclinical hypothyroidism^13^ can be independently associated to subclinical atherosclerosis and early cardiovascular disease measured by the Carotid Intima-Media Thickness (cIMT), a surrogate marker of atherosclerosis or early cardiovascular disease in its initial stages. Thus, we aimed to analyze cross-sectionally the association of diabetes, subclinical hypothyroidism and cIMT using data from the 3rd visit (2017‒2019) of the Brazilian Longitudinal Study of Adult Health (ELSA-Brazil). In this study, we hypothesized that the association of diabetes and subclinical hypothyroidism would result in early atherosclerotic disease reflected by a stronger association of cIMT with the subgroup of patients with both diabetes and subclinical hypothyroidism compared to individuals with only diabetes.

## Methods

ELSA-Brazil is a prospective cohort study designed to investigate cardiovascular disease and diabetes that enrolled 15.105 civil servants from five public education universities and one research institute located in the states of Bahia, Espírito Santo, Minas Gerais, Rio Grande do Sul, Rio de Janeiro and São Paulo.^14^, ^15^, ^16^ Briefly, all civil servants, active or retired, of these institutions, of both sexes, with ages between 35 and 74 years, were eligible for the study. Exclusion criteria were severe cognitive or communication impairment, intent to quit work in the near future and for those were retired, residence outside the corresponding metropolitan area. The study has been carried out in accordance with the Code of Ethics of the World Medical Association (Declaration of Helsinki) for studies involving humans. It was approved in the ethics committee of all six institutions. All participants provided signed informed consent.

This is a cross-sectional analysis using data from the third visit of ELSA-Brazil carried out between 2017‒2019.

### Definition of diabetes

A previously medical diagnosis of diabetes was classified when answering, “yes” to either “Have you been previously told by a physician that you had/have diabetes (sugar in the blood)?” or “Have you used medication for diabetes in the past 2-weeks?” Previously undiagnosed diabetes was classified based on laboratory values when reaching the threshold for fasting plasma glucose (FPG, < 126 mg/dL or ≥ 7.0 mmoL/L), 2-hour plasma glucose during the OGTT (2h PG < 140 mg/dL or ≥ 11.1 mmoL/L, or HbA1c (≥ 6.5%; ≥ 47.5 mmoL/moL).^17^

A 12-hour fasting blood sample was drawn in the morning soon after arrival at the research clinic, following standardized procedures for samples collection and processing.^17^^,^^18^ A standardized 75g Oral Glucose Tolerance test (OGTT) was performed in all participants without known diabetes utilizing an anhydrous glucose solution. Plasma glucose was measured by the hexokinase method (Roche Diagnostic, Manheim, Germany) HbA1c was measured by high pressure liquid chromatography (Bio-Rad Laboratories, Hercules, California).

### Thyroid function

TSH and FT4 levels were determined by a third generation immunoenzymatic assay (Roche Diagnostic, Manheim, Germany). Thyroid dysfunction was assessed by TSH and FT4 levels or by routine use of thyroid hormones or anti-thyroid medications, such as propylthiouracil or methimazole. Reference range for TSH was 0.4 to 4.0 mIU/L and for FT4 they were 0.93 ng/dL to > 1.7 ng/dL. Participants were placed in one of five groups based on TSH and FT4 levels (if TSH was altered) and their use of medications to treat thyroid disorders: clinical hyperthyroidism (low TSH, high FT4, or use of medications to treat hyperthyroidism), subclinical hyperthyroidism (low serum TSH, normal FT4, and no use of thyroid drugs), euthyroidism (normal TSH and no use of thyroid drugs), subclinical hypothyroidism (high TSH, normal FT4 levels, and no use of thyroid drugs), and clinical hypothyroidism (high TSH, low FT4, or use of levothyroxine.^19^

### Measurement of subclinical atherosclerosis and early cardiovascular disease

Carotid Intima-Media Thickness (cIMT) protocol was published earlier.^20^^,^^21^ In this paper, cIMT was defined as the average between the mean left and mean right carotid intima-media measurements. Briefly, cIMT was measured in the outer wall of a 1 cm long predefined carotid segment from 1 cm below the carotid bifurcation, during 3 cardiac cycles. Abnormal cIMT values were defined as those above the 75th percentile. cIMT, and also as a continuous variable. The evaluation of all images was centralized in the reading center in São Paulo after image quality assessment. An automatic software (MIA, Coralville, IA) was used to standardize the reading and interpretation of valid carotid scans.

### Other variables

Sociodemographic variables were sex; age (years); educational attainment (less than high school, high school and some college and at least complete college); self-reported race/skin color (White, Mixed, Black, Asian and Indigenous). Smoking and alcohol use were categorized as never, past or current.

Blood Pressure (BP) was measured three times using a validated Omron HEM 705CPINT oscillometric device. The average of the last two measurements was considered as the casual systolic or diastolic blood pressure. Hypertension was defined as use of medication to treat hypertension or systolic blood pressure ≥ 140 mm.Hg, or diastolic blood pressure ≥ 90 mm.Hg. Dyslipidemia was defined as LDL-cholesterol > 130 mg/dL or use of any lipid lowering medication in participants without diabetes. For men participants with diabetes if < 50 years we used the cut-off for dyslipidemia as > 100 mg/dL and if ≥ 50 years, the cut-off used was > 70 mg/dL. For women with diabetes, if < 56 years the cut-off used was < 100 mg/dL, and if ≥ 56 years, the cut-off was > 70 mg/dL. Self-reported leisure-time physical activity was classified as mild, moderate and vigorous.

### Statistical analysis

Categorical variables were expressed as numbers and proportions and analyzed using Chi-square test. Continuous variables were presented as mean (standard deviations) and compared using ANOVA if normal distributions; and as median (Interquartile range) and analyzed using nonparametric tests if non-normal distribution.

Linear regression models were built using no disease, subclinical hypothyroidism, diabetes and both diseases combined with cIMT as a continuous variable. Results were presented as Beta (95% Confidence Interval ‒95% CI) with crude, adjusted for sociodemographic variables (age strata, sex, education and self-reported race (Model 1) and in multivariable analysis adjusted for all variables on Model 1 more BMI, hypertension, dyslipidemia, smoking and physical activity (Model 2).

Logistic regression models were built using no disease, subclinical hypothyroidism, diabetes, and both diseases combined as independent variables with cIMT ≥ 75th percentile as the dependent one. Odds Ratio (OR) were presented with the same adjustments used in linear regression models.

To evaluate the possible additional additive effect of diabetes with subclinical hypothyroidism in the determination of a high cIMT we analyzed the interaction between diabetes and subclinical hypothyroidism with cIMT using CIMT as a continuous or a categorical variable. We considered as significant a p-value < 0.05. Data were analyzed using the Statistical Packages for Social Sciences SPSS version 25.0.

## Results

**Fig. 1:**
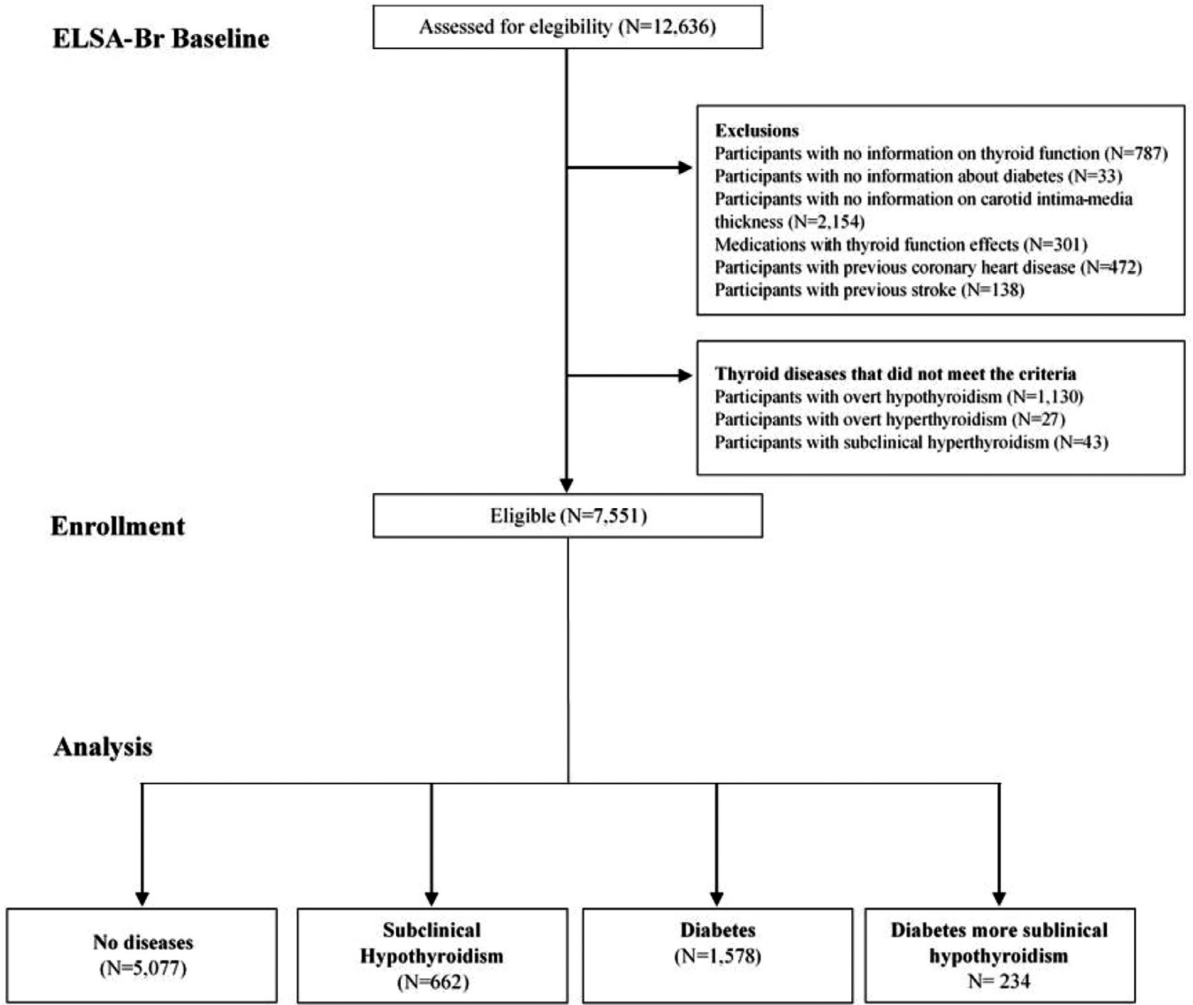
Participants included in the analysis.

In the analysis using subclinical hypothyroidism, of the 12,636 participants on the 3rd visit (2017‒2019), we excluded 787 participants with no information on thyroid function, 33 with no information about diabetes, 2,154 without information on cIMT, 301 using drugs that may alter thyroid function, 472 with previous coronary heart disease, 138 with the previous stroke, 1,130 with overt hypothyroidism, 27 with overt hyperthyroidism, 43 with subclinical hyperthyroidism remaining 7,751 participants: 5,077 without diabetes or subclinical hypothyroidism, 662 with subclinical hypothyroidism, 1,578 with diabetes, and 234 with both subclinical hypothyroidism and diabetes (Fig. 1). The time since the diagnosis of diabetes was around 16 years of age.

Table 1 shows the characteristics of the participants according to the presence of subclinical hypothyroidism, diabetes and both diseases. Progressive increases in age-strata, BMI, WC, and cIMT. (p for trend < 0.0001) were observed from the subgroup of no diseases to those with both diseases. The frequency of women was lower in the subgroup of diabetes or both diseases compared to other subgroups (p < 0.0001). The frequency of black self-reported race was lower in the subgroup of subclinical hypothyroidism compared to other groups (p < 0.0001); the frequency of hypertension was higher in patients with diabetes and both diseases while dyslipidemia was higher only in participants with both diseases (p < 0.0001 for both). Alcohol use decreased from the non-disease to both diseases subgroup (p < 0.0001). The frequency of vigorous physical activity was lower in the subgroups of diabetes or both diseases compared to others (p < 0.0001) while the frequency of current smoking was higher in individuals with diabetes, or no diseases compared to other subgroups.

**Table 1:** General and clinical characteristics of the sample according to the presence or not of subclinical hypothyroidism or diabetes.

Diagnosis of diabetes or subclinical hypothyroidism					
	No (n = 5,077)	Only Subclinical hypothyroidism (n = 662)	Only diabetes (n = 1,578)	Both (n = 234)	p
Age (years)	57.5 (8.4)*	59.5 (8.6)^*,†,‡^	60.6 (8.4)^*,†,§^	63.6 (8.6)^*,‡,§^	<0.0001
					p for trend <0.0001
Age strata (years)					<0.0001
35‒44	195 (3.8)^a^	17 (2.6)^a,b^	23 (1.5)^b,c^	1 (0.4)^c^	
45‒54	1,855 (36.5)^a^	189 (28.5)^b^	389 (24,7)^c^	34 (14.5)^c^	
55‒64	1,960 (38.6)^a^	265 (40.0)^a,b^	683 (43.3)^b^	91 (39)^a,b^	
65‒74	895 (17.6)^a^	163 (24.6)^b^	376 (23.8)^b^	82 (35.0)^c^	
75‒84	172 (3.4)^a^	28 (4.2)^b^	107 (6.8)^b^	26 (11.1)^c^	
Women (%)	2838a (55.9)^a^	340 (51.4)^b^	730 (43.6)^c^	100 (42.7)^c^	<0.0001
Self-reported race (%)					<0.0001
White	2,882 (57.4)^a^	420 (64.1)^b^	763 (48.8)^c^	126 (54.1)^a,c^	
Mixed	1,276^a^ (25.4)^a^	154 (23.5)^a^	421 (26.9)^a^	62 (26.6)^a^	
Black	703 (14)^a^	59 (9)^b^	299 (19.1)^c^	33 (14.2)^a,c^	
Asian	130 (2.6)^a^	14 (2.1)^a^	65 (4.2)^b^	7 (3.0)^a,b^	
Indigenous	31 (0.6)^a^	8 (1.2)^a,b^	16 (1.0)^a,b^	5 (2.1)^b^	
Education					<0.0001
Less than high-school	408 (8.0)^a^	66 (10.0)^a^	237 (15.0)^b^	39 (16.7)^b^	
High-school and some college	1,771 (34.9)^a^	221 (33.4)^a^	590 (37.4)^a,b^	102 (43.6)^b^	
Complete college or more	2,898 (57.1)^a^	375 (56.6)^a^	751 (47.6)^b^	93 (39.7)^c^	
Body Mass Index (BMI) kg/m^2^	27.1 (4.6)*	27.6 (5.0)^†^	29.6 (5)^*,†^	29.3 (4.9)^†,*^	<0.0001
					p for trend <0.0001
Waist circumference (cm)	93.4 (12.4)*	94.4 (12.6)^†^	101.8 (12.8)^*,†^	101.7 (12.5)^*,†^	<0.0001
					p for trend <0.0001
Hypertension (%)	1,849 (36.5)^a^	251 (38.1)^a^	1,004 (63.9)^b^	143 (61.4)^b^	<0.0001
HbA1c levels	5.41 (0.41)	5.43 (0.36)	6.82 (1.71)	6.87 (1.54)	<0.0001
TSH levels (IU/L)	2.07 (0.83)	5.30 (1.43)	2.09 (0.84)	5.43 (1.6b0)	<0.0001
TSH levels ≥10 IU/L	0^a^	9^b^	0^a^	4^b^	<0.0001
Dyslipidemia (%)	2467 (48.6)^a^	350 (52.9)^b^	1441 (91.3)^c^	229 (97.9)^c^	p < 0.0001
Smoking (%)					<0.0001
Never	3158 (62.4)^a^	427 (64.8)^a^	826 (52.5)^b^	139 (59.4)^a^	
Past	1,388 (27.4)^a^	184 (27.9)^a^	575 (36.6)^b^	85 (36.3)^b^	
Current	517 (10.2)^a^	48 (7.3)^b^	171 (10.9)^a^	10 (4.3)^b^	
Alcohol use (%)	3,479 (68.7)^a^	438 (66.3)^a,b^	962 (63.3)^b,c^	134(57.5)^c^	<0.0001
Physical activity (%)					<0.0001
Mild	3,363 (66.4)^a^	442 (67.0)^a^	1,146 (72.9)^b^	167 (71.4)^a,b^	
Moderate	1,129 (22.3)^a^	156 (23.6)^a^	317 (20.2)^a^	51 (21.8)^a^	
Vigorous	569 (11.2)^a^	62 (9.4)^a,b^	108 (6.9)^c^	16 (6.8)^b,c^	
Intima-media thickness (mm)	0.66 (0.13)*	0.67 (0.13)^†^	0.71 (0.16)^*,†^	0.73 (0.20)^*,†^	<0.0001
					p for trend <0.0001
Intima-media thickness > P75%	986 (19.4)^a^	135 (20.4)^a^	547 (34.7)^b^	86 (36.8)^b^	<0.0001

IQR, Interquartile range; SCHypo, Subclinical hypothyroidism.

For Age: *participants with no disease ≠ *SCHypo, *diabetes and *both diseases, for all p < 0.0001; †participants with SCHypo ≠ †diabetes, p = 0.02; ‡SCHypo ≠ ‡both diseases, p < 0.0001; §diabetes ≠ both diseases, p < 0.0001.

For BMI: *No disease ≠ diabetes and both diseases, p < 0.0001 for both; †SCHypo ≠ †diabetes or †both diseases, p < 0.0001 for both.

For WC: *participants with no disease ≠ diabetes and both diseases, p < 0.0001 for both; †SCHypo ≠ †diabetes or †both diseases, p < 0.0001 for both.

For cIMT: †,*participants with no disease ≠ diabetes and both diseases, p < 0.0001 for both; †SCHypo ≠ †diabetes or †both diseases, p < 0.0001 for both.

Table 2 shows the association of subclinical hypothyroidism, diabetes and both diseases with cIMT. Linear regression models showed an association of the subgroup of diabetes with cIMT (Beta = 0.019; 95% CI 0.012 to 0.027, p < 0.0001) and also of the subgroup with both diseases with cIMT (Beta = 0.03; 95% CI 0.010‒0.047, p < 0.0001). No other associations were found.

**Table 2:** Odds Ratio (95% Confidence Interval) of the association of diabetes, subclinical thyroid disorders, and both diseases with carotid intima-media thickness cIMT > P75%.

	Crude	Model 1	Model 2
CIMT > P75%, subclinical hypothyroidism and diabetes			
No disease	1.0 (Reference)	1.0 (Reference)	1.0 (Reference)
Only subclinical hypothyroidism	1.05 (0.87‒1.30)	0.92 (0.75‒1.14)	0.93 (0.75‒1.16)
Only diabetes	2.20 (1.94‒2.49)	1.72 (1.50‒1.96)	1.49 (1.30‒1.71)
Both diabetes and subclinical hypothyroidism	2.41 (1.83‒3.17)	1.47 (1.10‒1.97)	1.33 (0.99‒1.70)
CIMT (continuous), subclinical hypothyroidism and diabetes			
	0 (Reference)	0 (Reference)	0 (Reference)
Only subclinical hypothyroidism	0.008 (-0.003 to 0.02)	-0.002 (-0.012 to 0.009)	0.000 (-0.011 to 0.01)
	p = 0.16	p = 0.77	p = 0.96
Only diabetes	0.051 (0.043 to 0.059)	0.030 (0.023 to 0.038)	0.019 (0.012 to 0.027)
	p < 0.0001	p < 0.0001	p < 0.0001
Both diabetes and subclinical hypothyroidism	0.073 (0.055 to 0.092)	0.036 (0.018 to 0.053)	0.03 (0.010 to 0.047)
	p < 0.0001	p < 0.0001	p < 0.0001

cIMT, Carotid Intima-Media Thickness; Model 1, adjusted by age, sex, self-reported race, and education; Model 2, adjusted by all variables in model 1 more Body-Mass Index (BMI), hypertension, dyslipidemia, smoking, and self-reported physical activity at leisure.

We also analyzed whether there was an interaction between diabetes and subclinical hypothyroidism on cIMT values. We found neither significant interaction terms between diabetes and subclinical hypothyroidism for continuous cIMT values (p = 0.29) nor categorical cIMT > 75th percentile (p = 0.92).

## Discussion

In the present study, the main cardiovascular risk factor consistently associated with an augmented cIMT was diabetes. In the subgroup with both diabetes and subclinical hypothyroidism, there was an association with cIMT values only in the linear regression models, but no additive effect in patients with both diseases was detected.

Some reasons may explain our findings that did not show any additive effect of both subclinical hypothyroidism and diabetes over cIMT in the entire sample. The mean age in the 3rd visit of the ELSA-Brazil study is around 59 years, and most of the participants of the cohort are less than 60 years-old even 9 years after baseline. Therefore, participants are still relatively young while the prevalence of diabetes and subclinical hypothyroidism increases with age. Moreover, ELSA-Brazil is an occupational cohort study with a health worker bias effect.

Previous publications in the ELSA-Brazil confirmed the association of cIMT and subclinical hypothyroidism^22^ as well as a recent meta-analysis did.^13^ However, we could not find any study that compared subclinical atherosclerosis or early cardiovascular disease among individuals with both type 2 diabetes and subclinical hypothyroidism. Only one small study evaluated the impact of Hashimoto's thyroiditis and type 1 diabetes in 20 patients with both diseases, 30 patients with only diabetes, and 20 healthy controls. It was shown that both groups of participants with diabetes (with and without Hashimoto thyroiditis) presented higher cIMT values compared to the control group. However, no difference in cIMT values was detected between participants with only diabetes and with both diseases.^23^ The negative results may also be explained by the inclusion of adolescents and young adults with a mean age of around 17 years of age which hampers any association with cIMT. Although these results are similar to ours, there is a very important difference since they included young participants with type 1 diabetes compared to our middle-aged and older patients with type 2 diabetes.

Our analysis of a possible interaction effect of diabetes and subclinical hypothyroidism over cIMT was based on previous studies that evaluated the association of subclinical thyroid dysfunction and diabetes with complications of diabetes including previous findings of the ELSA-Brazil study. In a previous analysis of the ELSA-Brazil baseline, the additive effect between diabetes and subclinical hypothyroidism impacted the lower cardiac autonomic control in participants with both diseases. In the same study, we found a borderline significant interaction term for the association of participants with diabetes and subclinical hypothyroidism with heart rate variability.^24^

Several studies evaluated the association of participants with diabetes and subclinical hypothyroidism with more complications of diabetes in patients with both diseases with heterogeneous findings. A meta-analysis of Han et al. reported the high impact of the association between diabetes and subclinical hypothyroidism on the number of diabetes complications. Subclinical hypothyroidism was associated with a higher prevalence of overall risk of diabetes, diabetic nephropathy, retinopathy, peripheral arterial disease, and peripheral neuropathy.^6^ In addition, Mansournia et al. reported a cross-sectional association of subclinical hypothyroidism with chronic kidney disease in a sample of patients diagnosed with diabetes.^10^ In the study of Reddy et al. performed on 500 participants with subclinical hypothyroidism and type 2 diabetes there was an association in participants with both diseases with a higher frequency of retinopathy.^25^ Jia et al. also reported in a cross-sectional analysis of patients with type 2 diabetes and subclinical hypothyroidism, higher frequencies of CHD.^11^ Other small studies reported similar results.^26^, ^27^, ^28^ Contrasting with these previous findings, Sharma et al. reported in a sample of patients with both diabetes and subclinical hypothyroidism that glycemic control was similar to that of patients with only diabetes.^29^ Mehalingam et al. also reported in a small sample with diabetes that prevalent thyroid dysfunction was not associated with a higher degree of diabetic complications.^30^ Therefore, although heterogeneous findings, most studies reported an association between diabetes and subclinical hypothyroidism with a greater frequency of diabetic complications.

The biological plausibility for a possible association between diabetes, subclinical and overt hypothyroidism is explained by hyperglycemia linked to impaired insulin secretion and also with insulin resistance.^3^ On the other hand, Thyroid Hormones (TH) may influence the balance of glucose and insulin homeostasis.^3^ In addition, subclinical hypothyroidism may be associated with higher glucose levels^31^ and at the same time with lesser use of peripheral glucose^32^ with a decrease both in glucose oxidation and glycogen synthesis^33^ and also insulin resistance.^34^^,^^35^ Although, subclinical hyperthyroidism may be associated with gluconeogenesis in the liver^36^ and increased insulin degradation,^37^ and insulin resistance^34^^,^^38^ in this analysis it was impossible to evaluate subclinical hyperthyroidism because of the small number of participants with both diabetes and subclinical hyperthyroidism (n = 11).

The present study has some strengths. ELSA-Brazil used centralized protocols to train the research team under strict quality control. Diagnosis of diabetes was very comprehensive including fasting plasma glucose, an oral glucose tolerance test and also glycate hemoglobin, previous medical history of diabetes, and use of medication to treat diabetes. No previous study evaluated the combined effect of both diabetes and subclinical thyroid diseases using cIMT as the outcome in a highly admixed sample from a middle-income country as Brazil. Also, important limitations should point out here. The number of cases of both diabetes and subclinical thyroid diseases is small which limits our power to conduct a separate analysis in this subgroup. This study is a cross-sectional analysis that only can evaluate association but not causality. In addition, there is scarce data on the association of both diabetes and subclinical hypothyroidism using CIMT as an endpoint which difficult comparisons of our data with other studies.

In conclusion, our results confirmed the association of diabetes with cIMT. However, we do not show any interaction effect of subclinical hypothyroidism and diabetes with early cardiovascular disease measured by cIMT.

## Funding sources

Ministry of Health: grants BA405551/2015-0; MG405552/2015/7; ES405543/2015-8; RJ405544/2015-4; RG405545/2015-0; SP 405547/2015-3; FAPESP 2019/17213-6.

## Conflicts of interest

The authors declare no conflicts of interest.
